# COVID-19 MORTALITY RATES AND CHANGES IN ANTI-DEMENTIA AND PSYCHOTROPIC MEDICATION USE AMONG NURSING HOME RESIDENTS

**DOI:** 10.1093/geroni/igae098.2837

**Published:** 2024-12-31

**Authors:** Alison Rataj, Matthew Alcusky, Brian Ott, Jonggyu Baek, Shiwei Liang, Kate Lapane

**Affiliations:** University of Massachusetts Boston, Durham, New Hampshire, United States; University of Massachusetts Chan Medical School, Worcester, Massachusetts, United States; Brown University, Providence, Rhode Island, United States; University of Massachusetts Chan Medical School, Worcester, Massachusetts, United States; University of Massachusetts Chan Medical School, Worcester, Massachusetts, United States; University of Massachusetts Chan Medical School, Worcester, Massachusetts, United States

## Abstract

Objective. We examined the relationship between nursing home (NH) COVID-19 mortality rates and changes in antidementia and psychotropic medication initiation before and during the pandemic and explored the influence of staffing and resident factors. Methods. Changes in medication initiation were collected through a novel, nationally representative cross-sectional Dementia Treatment Survey (2022) of NH Directors of Nursing. Outcome measures were created by collapsing a 5-point Likert scale contrasting less/about the same vs. more initiation and less versus more/about the same initiation. NH’s peak monthly COVID-19 death rate per 1,000 residents (05/2020-12/2022) was calculated from the CMS COVID-19 Nursing Home data file. Covariates were drawn from NH Compare, Provider of Service, Master Beneficiary Summary, Minimum Data Set 3.0, and Medicare administrative claims files. Adjusted odds ratios (aORs) were estimated from multivariable logistic models. Results. NHs with higher COVID-19 death rates at the peak of the pandemic were less likely to report changes in antidementia medications (aORdecrease: 0.38, 95% CI: 0.15-0.94; aORIncrease: 0.27, 95% CI: 0.08-0.85), but no association with psychotropic medication initiation was observed. NH’s that reported increased resident behavior problems during the pandemic had higher odds of psychotropic initiation (aOR=11.25, CI=3.86-32.83). Discussion. Increased COVID-19 mortality rates were not associated with changes in antidementia medication use during compared to before the pandemic. Increased behavior changes may be due to consequences of COVID-19 (i.e., social isolation); our findings indicate that increased behavior changes were associated with more psychotropic drug use.

